# Five-Year Outcomes of First-Generation iStent Versus Hydrus Microstent Implantation Combined with Phacoemulsification in Patients with Open-Angle Glaucoma: A Prospective Non-Randomized Comparative Study

**DOI:** 10.3390/jcm15135076

**Published:** 2026-06-29

**Authors:** Joanna Jabłońska, Katarzyna Lewczuk, Karolina Krix-Jachym, Natalia Błagun, Marek Rękas

**Affiliations:** Ophthalmology Department, Military Institute of Medicine—National Research Institute, Szaserów Street 128, 04-141 Warsaw, Poland

**Keywords:** iStent, Hydrus Microstent, open-angle glaucoma, micro-invasive glaucoma surgery, phacoemulsification, 5-year follow-up

## Abstract

**Background**: This study assessed the 5-year clinical outcomes of phacoemulsification combined with implantation of either the first-generation iStent Trabecular Micro-Bypass or the Hydrus Microstent in eyes with open-angle glaucoma. **Methods**: In this prospective, non-randomized comparative study, 65 eyes of 65 patients underwent combined cataract and micro-invasive glaucoma surgery with either iStent or Hydrus implantation. Intraocular pressure (IOP), number of glaucoma medications, best-corrected visual acuity (BCVA), surgical success, postoperative complications, and subsequent glaucoma procedures were analyzed over a 60-month follow-up. **Results**: At 60 months, outcome data were available for 47 eyes (72.3%), including 25 eyes in the iStent group and 22 eyes in the Hydrus group. Baseline characteristics did not differ significantly between groups. Mean IOP at 60 months was similar after iStent and Hydrus implantation (16.7 ± 1.8 mmHg vs. 16.5 ± 1.9 mmHg, respectively). The mean number of glaucoma medications decreased from 1.86 ± 0.94 to 1.36 ± 1.08 in the iStent group and from 1.60 ± 0.72 to 0.36 ± 0.49 in the Hydrus group, with significantly fewer medications required after Hydrus implantation at 60 months. Medication-free complete surgical success using the IOP ≤ 18 mmHg criterion was achieved in 20.0% of iStent-treated eyes and 63.6% of Hydrus-treated eyes. No eye underwent additional glaucoma surgery or selective laser trabeculoplasty during follow-up. **Conclusions**: In this prospective non-randomized comparative cohort, both procedures provided comparable long-term treated IOP control when combined with phacoemulsification. Hydrus implantation was associated with a greater medication-sparing effect and a higher proportion of medication-free complete surgical success at 5 years; however, these findings should be interpreted in the context of the non-randomized design and available-case follow-up.

## 1. Introduction

Primary open-angle glaucoma is a progressive optic neuropathy in which lowering intraocular pressure remains the only established modifiable strategy for slowing disease progression [1]. In daily clinical practice, treatment often begins with topical therapy or laser trabeculoplasty; however, long-term control may be compromised by poor adherence, local intolerance, ocular surface disease, and the cumulative burden of chronic medication use [1]. These limitations have contributed to the development and wider adoption of micro-invasive glaucoma surgery (MIGS), particularly for patients with mild-to-moderate open-angle glaucoma undergoing cataract surgery. Compared with conventional filtering procedures, MIGS is intended to provide a more favorable safety profile while reducing intraocular pressure and/or the need for topical medications [2].

Trabecular MIGS procedures target the conventional aqueous outflow pathway and have become an important option in combined cataract and glaucoma surgery. The first-generation iStent Trabecular Micro-Bypass was one of the first trabecular bypass implants assessed in randomized trials as an adjunct to phacoemulsification [3]. The Hydrus Microstent acts as an intracanalicular scaffold within Schlemm’s canal and has been shown to improve outflow and reduce treatment burden when implanted at the time of cataract surgery in eyes with mild-to-moderate primary open-angle glaucoma [4]. Although both devices are used in a similar clinical setting, their mechanisms of action and extent of canal-based treatment differ, which may influence long-term medication requirements and surgical success.

We previously reported 24-month outcomes from a prospective non-randomized comparative cohort of eyes treated with phacoemulsification combined with either first-generation iStent or Hydrus Microstent implantation. In that analysis, both procedures showed a favorable safety profile and effective intraocular pressure control, while the reduction in glaucoma medication use was greater in the Hydrus group [5]. Whether these differences persist over a longer follow-up remains clinically relevant, because sustained medication reduction may improve adherence, ocular surface comfort, and overall treatment burden.

The aim of the present study was to compare five-year outcomes of first-generation iStent and Hydrus Microstent implantation combined with phacoemulsification, focusing on intraocular pressure control, glaucoma medication burden, surgical success, visual function, safety, and the need for additional glaucoma procedures.

## 2. Materials and Methods

This prospective, non-randomized comparative study analyzed 65 eyes of 65 patients with open-angle glaucoma who underwent combined cataract and glaucoma surgery. Phacoemulsification was performed together with implantation of either the first-generation iStent Trabecular Micro-Bypass or the Hydrus Microstent. All procedures and follow-up examinations were carried out at the Department of Ophthalmology, Military Institute of Medicine—National Research Institute in Warsaw, Poland. The current report presents the 5-year follow-up of the cohort previously described at 24 months. Patients were enrolled between January 2016 and December 2016 and were followed prospectively for 60 months after surgery. The primary 60-month follow-up period was completed in December 2021. The study was conducted in accordance with the Declaration of Helsinki and was approved by the Bioethics Committee of the Military Institute of Medicine in Warsaw, Poland (Resolution No. 75/WIM/2015; approval date: 16 December 2015). All patients provided written informed consent before surgery.

Eligible patients had visually significant cataract and open-angle glaucoma requiring IOP-lowering therapy. Patients were included when glaucoma progression was documented despite topical treatment or when medication intolerance or poor adherence made long-term topical therapy problematic. Disease progression was evaluated using standard automated perimetry with the Humphrey Field Analyzer (HFA, Carl Zeiss Meditec AG, Jena, Germany) and the SITA Standard 24-2 strategy. Exclusion criteria were refusal to participate, narrow-angle or angle-closure glaucoma, advanced glaucoma, previous glaucoma surgery or laser trabeculoplasty, treatment with more than four IOP-lowering medications, severe proliferative diabetic retinopathy, corneal opacity limiting reliable assessment, advanced age-related macular degeneration, and any other ocular disease likely to interfere with postoperative visual outcomes or follow-up evaluation.

Before surgery, the following data were recorded: demographic characteristics, glaucoma diagnosis, operated eye, number of glaucoma medications, best-corrected visual acuity (BCVA), intraocular pressure (IOP), slit-lamp findings, gonioscopic assessment, fundus examination, central corneal thickness (CCT), cup-to-disc ratio, and visual field indices, including mean deviation (MD) and pattern standard deviation (PSD). IOP was measured by Goldmann applanation tonometry (Haag-Streit AG, Köniz, Switzerland), and BCVA was assessed using a Snellen chart. Gonioscopy was performed with a Goldmann three-mirror lens (Ocular Instruments Inc., Bellevue, WA, USA), and the anterior chamber angle was graded according to the Shaffer classification. Visual field testing was performed with the Humphrey Field Analyzer using the SITA Standard 24-2 algorithm.

Postoperative visits included assessment of BCVA, IOP, number of glaucoma medications, anterior segment findings on slit-lamp examination, and fundus examination. For this long-term analysis, data were evaluated at baseline and at 12, 24, 36, 48, and 60 months after surgery. Analyses were based on available cases at each scheduled follow-up visit. Eyes without 60-month data were classified as lost to follow-up when no subsequent clinical visits were available. One patient died after completing the 18-month visit. During the 5-year observation period, no eye underwent additional glaucoma surgery or selective laser trabeculoplasty.

All operations were performed by the same surgeon under local anesthesia. In each case, cataract surgery was completed first, using standard phacoemulsification followed by posterior chamber intraocular lens implantation into the capsular bag. After completion of the cataract procedure, the assigned trabecular MIGS implant was inserted ab interno under gonioscopic visualization.

For Hydrus Microstent (Ivantis Inc., Irvine, CA, USA) implantation, the nasal angle was visualized with a gonioscopic lens. The delivery system was introduced through the temporal clear corneal incision used for phacoemulsification. After the trabecular meshwork was entered with the cannula tip, the Hydrus Microstent was advanced into Schlemm’s canal, with the inlet remaining visible in the anterior chamber.

For first-generation iStent Trabecular Micro-Bypass (Glaukos Corp., Aliso Viejo, CA, USA) implantation, the applicator was passed through the same temporal clear corneal incision and directed toward the nasal angle under gonioscopic control. The stent was implanted through the trabecular meshwork into Schlemm’s canal, and its position was verified intraoperatively.

At the end of surgery, the viscoelastic material was aspirated from the anterior chamber. Postoperatively, all patients received topical antibiotic and anti-inflammatory therapy according to the standard institutional protocol. Glaucoma medications were stopped after surgery and restarted when the target IOP was not achieved, based on clinical assessment and European Glaucoma Society recommendations [1].

The primary outcomes were IOP and the number of glaucoma medications during the 5-year follow-up. Secondary outcomes included BCVA, surgical success, visual field indices, postoperative complications, and the need for any additional glaucoma procedure. Surgical success was evaluated using two IOP cut-off values: ≤18 mmHg and ≤15 mmHg. Complete success was defined as achievement of the specified IOP threshold without glaucoma medications and without subsequent glaucoma surgery or selective laser trabeculoplasty. Qualified success was defined as achievement of the specified IOP threshold with no more than two glaucoma medications and without subsequent glaucoma surgery or selective laser trabeculoplasty.

Additional efficacy endpoints included the proportion of medication-free eyes, the proportion of eyes with a reduction of at least one or at least two glaucoma medications from baseline, and the proportion of eyes achieving at least 20% IOP reduction from baseline. Visual field outcomes were assessed using MD and PSD values when reliable visual field data were available. For the 5-year analysis, baseline and 60-month MD values were compared, and eyes with MD deterioration of ≥2.5 dB were additionally identified.

Safety outcomes included blood cells in the anterior chamber, IOP increase of ≥10 mmHg from baseline, peripheral anterior synechiae, hypotony defined as IOP < 6 mmHg, corneal edema, implant migration or dislocation, and the need for further glaucoma intervention, including glaucoma surgery or selective laser trabeculoplasty.

Continuous variables were summarized as mean and standard deviation (SD), and categorical variables as numbers and percentages. Distribution normality was assessed with the Shapiro–Wilk test. Between-group comparisons for continuous variables were performed using Student’s *t*-test or the Mann–Whitney U test, according to data distribution. Within-group changes from baseline were evaluated with the paired Student’s *t*-test or the Wilcoxon signed-rank test, as appropriate. Categorical variables were compared using Fisher’s exact test or the chi-square test.

All analyses were conducted using available cases at each follow-up time point, with the number of eyes included in each analysis reported. To address potential attrition bias, baseline characteristics of eyes with available 60-month data were compared with those of eyes without 60-month follow-up data. An additional exploratory post hoc analysis was performed to assess IOP change at 60 months according to baseline IOP. Eyes with available 60-month data were stratified into those with baseline IOP > 18 mmHg and those with baseline IOP ≤ 18 mmHg. A *p*-value < 0.05 was considered statistically significant. Statistical analyses were performed using Statistica software, version 13.3 (TIBCO Software Inc., Palo Alto, CA, USA).

## 3. Results

Sixty-five eyes of 65 patients were included in the study. Of these, 35 eyes underwent phacoemulsification combined with first-generation iStent Trabecular Micro-Bypass implantation, and 30 eyes underwent phacoemulsification combined with Hydrus Microstent implantation. Baseline demographic and clinical characteristics are summarized in Table 1.

Baseline characteristics were comparable between the two treatment groups. No statistically significant differences were found in sex distribution, operated eye, age, baseline BCVA, IOP, number of glaucoma medications, CCT, MD, or PSD.

**Figure 1:**
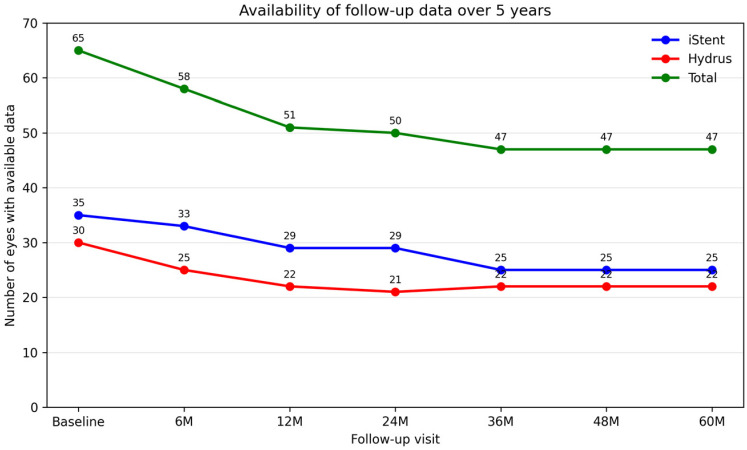
Availability of follow-up data over the 5-year observation period. The figure shows the number of eyes with available follow-up data at each postoperative time point in the iStent and Hydrus groups.

Five-year IOP, medication, and BCVA data were available for 47 of 65 eyes (72.3%), including 25 of 35 eyes (71.4%) in the iStent group and 22 of 30 eyes (73.3%) in the Hydrus group. The availability of follow-up data over the 5-year observation period is shown in Figure 1. Seventeen eyes were lost to follow-up because no further visits were recorded, and one patient died after the 18-month visit. Baseline characteristics of eyes with available 60-month data and those without 60-month follow-up data were compared to assess potential attrition bias and are presented in Supplementary Table S1. A CONSORT-style flow diagram summarizing patient allocation, follow-up availability, and inclusion in the 60-month analysis is provided as Supplementary Figure S2. No eye required additional glaucoma surgery or selective laser trabeculoplasty during the 5-year follow-up period.

### 3.1. Intraocular Pressure

Mean IOP values at baseline and at 12, 24, 36, 48, and 60 months after surgery are presented in Table 2.

At baseline, mean IOP was 16.1 ± 3.2 mmHg in the iStent group and 16.4 ± 2.2 mmHg in the Hydrus group. At the 60-month visit, mean IOP was 16.7 ± 1.8 mmHg and 16.5 ± 1.9 mmHg, respectively. Mean IOP did not differ significantly between the iStent and Hydrus groups at any postoperative time point. In the iStent group, a significant reduction from baseline was observed at 12 months, with a mean decrease of 1.4 mmHg (*p* = 0.009), but this effect was not sustained at later visits. At 60 months, the mean change from baseline was −0.1 mmHg in the iStent group and 0.0 mmHg in the Hydrus group. No significant within-group reduction in IOP from baseline to 60 months was observed in either treatment group.

In the additional exploratory post hoc analysis stratified by baseline IOP, 10 eyes had baseline IOP > 18 mmHg and available 60-month data. In this subgroup, mean IOP decreased from 20.4 ± 1.3 mmHg at baseline to 17.2 ± 1.3 mmHg at 60 months, corresponding to a mean reduction of 3.2 ± 1.9 mmHg. In contrast, among 37 eyes with baseline IOP ≤ 18 mmHg and available 60-month data, mean IOP changed from 15.5 ± 2.1 mmHg at baseline to 16.4 ± 1.9 mmHg at 60 months. These findings indicate that eyes with higher treated baseline IOP showed greater numerical IOP reduction, whereas eyes with lower baseline IOP primarily maintained similar treated IOP over long-term follow-up.

**Figure 2:**
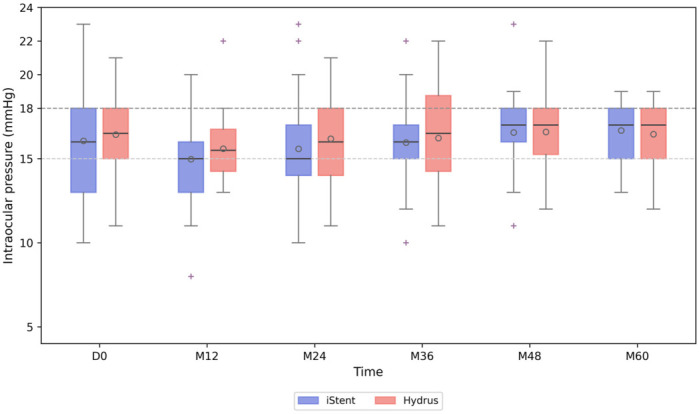
IOP values for the iStent and Hydrus groups during the 5-year follow-up. Boxes show the median and interquartile range; whiskers indicate data spread; hollow circles indicate mean values; crosses indicate outliers; dashed horizontal lines indicate IOP levels of 15 and 18 mmHg.

The distribution of IOP values over the 5-year follow-up is shown in Figure 2.

### 3.2. Glaucoma Medications

The number of glaucoma medications decreased after surgery in both treatment groups. At baseline, the mean medication burden was 1.86 ± 0.94 medications in the iStent group and 1.60 ± 0.72 medications in the Hydrus group, with no significant between-group difference. The number of glaucoma medications at each follow-up visit is presented in Table 3.

**Figure 3:**
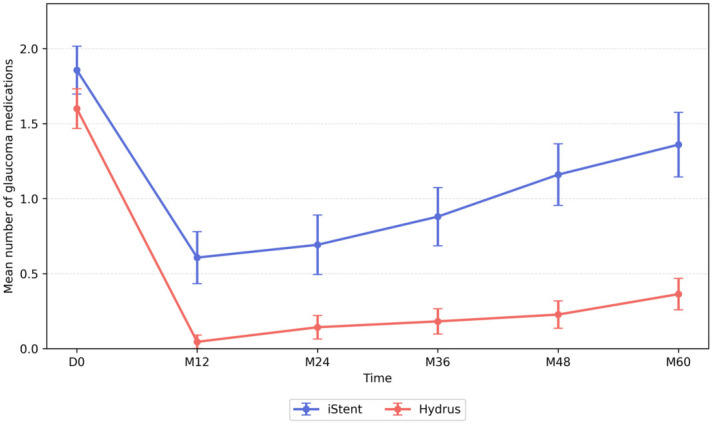
Mean number of glaucoma medications in the iStent and Hydrus groups during the 5-year follow-up. Error bars indicate the standard error of the mean.

At 60 months, the mean number of glaucoma medications was 1.36 ± 1.08 in the iStent group and 0.36 ± 0.49 in the Hydrus group. From 12 months onward, Hydrus-treated eyes required significantly fewer glaucoma medications than iStent-treated eyes at every postoperative follow-up visit. The longitudinal change in medication burden is shown in Figure 3.

At 60 months, medication-free status was achieved in 6 eyes (24.0%) in the iStent group and 14 eyes (63.6%) in the Hydrus group (*p* = 0.009). A reduction of at least one glaucoma medication from baseline was observed in 13 eyes (52.0%) and 18 eyes (81.8%), respectively (*p* = 0.063). A reduction of at least two medications was observed in 6 eyes (24.0%) and 8 eyes (36.4%), respectively (*p* = 0.524).

### 3.3. Surgical Success

Surgical success at 60 months was assessed using the predefined IOP thresholds of ≤18 mmHg and ≤15 mmHg. Complete and qualified success rates, together with additional clinical outcomes, are presented in Table 4.

**Figure 4:**
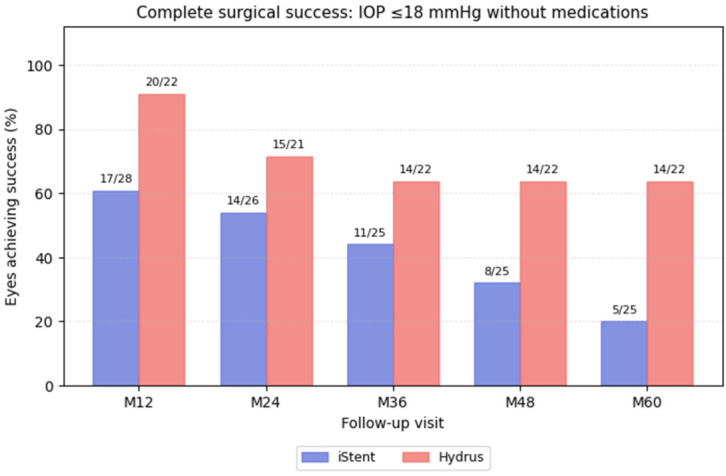
Complete surgical success according to the IOP ≤ 18 mmHg criterion during the 5-year follow-up. Complete success was defined as IOP ≤ 18 mmHg without glaucoma medications and without additional glaucoma surgery or SLT. Values above the bars indicate the number of eyes meeting the criterion among eyes with available data at each visit.

At 60 months, complete surgical success using the IOP ≤ 18 mmHg threshold was achieved in 5 eyes (20.0%) in the iStent group and 14 eyes (63.6%) in the Hydrus group (*p* = 0.003). Qualified surgical success using the same IOP threshold was achieved in 17 eyes (68.0%) and 20 eyes (90.9%), respectively (*p* = 0.079). The proportion of eyes achieving complete surgical success according to the IOP ≤ 18 mmHg criterion over the 5-year follow-up is shown in Figure 4.

**Figure 5:**
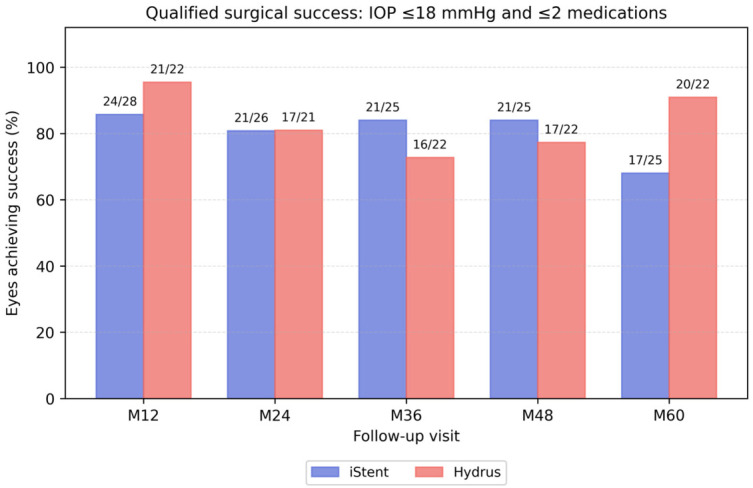
Qualified surgical success according to the IOP ≤ 18 mmHg criterion during the 5-year follow-up. Qualified success was defined as IOP ≤ 18 mmHg with no more than two glaucoma medications and without additional glaucoma surgery or SLT. Values above the bars indicate the number of eyes meeting the criterion among eyes with available data at each visit.

The proportion of eyes achieving qualified surgical success according to the IOP ≤ 18 mmHg criterion over the 5-year follow-up is shown in Figure 5.

Using the more stringent IOP ≤ 15 mmHg threshold, complete surgical success at 60 months was achieved in 1 eye (4.0%) in the iStent group and 5 eyes (22.7%) in the Hydrus group (*p* = 0.085). Qualified surgical success according to the IOP ≤ 15 mmHg criterion was achieved in 6 eyes (24.0%) and 8 eyes (36.4%), respectively (*p* = 0.524).

At 60 months, IOP ≤ 18 mmHg was achieved in 20 eyes (80.0%) in the iStent group and 20 eyes (90.9%) in the Hydrus group (*p* = 0.423). IOP ≤ 15 mmHg was achieved in 7 eyes (28.0%) and 8 eyes (36.4%), respectively (*p* = 0.755). An IOP reduction of at least 20% from baseline was observed in 2 eyes (8.0%) in the iStent group and 1 eye (4.5%) in the Hydrus group (*p* = 1.000).

### 3.4. Visual Field Outcomes

Reliable visual field data at both baseline and 60 months were available for a limited number of eyes. Therefore, the visual field analysis was exploratory and was limited to eyes with complete baseline and 60-month MD data; baseline and 60-month MD values were compared to explore functional outcomes over the 5-year follow-up. Eyes with MD deterioration of ≥2.5 dB were additionally identified. Individual baseline and 60-month MD values are presented in Supplementary Figure S1.

### 3.5. Safety

No intraoperative complications were recorded in either treatment group. The most frequent early postoperative finding was blood cells in the anterior chamber, observed in 12 eyes (40.0%) in the Hydrus group and in no eyes in the iStent group. All cases resolved spontaneously and did not require additional intervention.

A transient IOP increase of ≥10 mmHg from baseline occurred in 4 eyes (11.4%) in the iStent group and in 2 eyes (6.7%) in the Hydrus group. Peripheral anterior synechiae were observed in 6 eyes (20.0%) in the Hydrus group and were not observed in the iStent group. One case of transient hypotony and three cases of corneal edema occurred in the Hydrus group. None of these events required surgical management.

Most postoperative findings were transient and self-limiting. Although anterior chamber blood cells and peripheral anterior synechiae were observed more frequently in the Hydrus group, these findings did not lead to additional surgical intervention or selective laser trabeculoplasty during follow-up.

During the 5-year follow-up, no eye in either group underwent additional glaucoma surgery or selective laser trabeculoplasty. Safety outcomes and postoperative complications are summarized in Table 5.

## 4. Discussion

The present study provides 5-year comparative follow-up data for first-generation iStent Trabecular Micro-Bypass and Hydrus Microstent implantation performed in combination with phacoemulsification in eyes with open-angle glaucoma. The main finding was that both procedures were associated with comparable treated IOP at 60 months, whereas Hydrus-treated eyes required significantly fewer glaucoma medications. Thus, the principal between-group difference was the medication-sparing effect rather than a greater IOP-lowering effect. At the final follow-up visit, mean IOP was 16.7 ± 1.8 mmHg in the iStent group and 16.5 ± 1.9 mmHg in the Hydrus group, while the mean number of medications was 1.36 ± 1.08 and 0.36 ± 0.49, respectively. Accordingly, medication-free complete surgical success using the IOP ≤ 18 mmHg criterion was achieved more frequently after Hydrus implantation. Importantly, no eye in either group required additional glaucoma surgery or selective laser trabeculoplasty during the 5-year follow-up.

These results extend our previously published 24-month prospective non-randomized comparative study based on the same cohort [5]. In that earlier analysis, both procedures showed favorable safety and efficacy, with a greater reduction in medication burden in the Hydrus group [5]. The present 5-year findings suggest that this difference in medication dependence persists over longer follow-up, despite similar final treated IOP values. This distinction is clinically relevant because reducing topical treatment is an important goal of MIGS, particularly in patients with mild-to-moderate glaucoma, ocular surface disease, medication intolerance, or adherence difficulties.

The comparable IOP values at 60 months should therefore be interpreted together with postoperative medication use. Hydrus-treated eyes achieved a similar final IOP while requiring fewer medications, indicating a stronger medication-sparing effect rather than a clearly greater IOP-lowering effect. This distinction is important in long-term real-world follow-up, where clinicians adjust treatment to maintain individualized target IOP. As a result, final treated IOP may become similar between groups even when the number of medications needed to achieve that pressure differs.

The relatively low baseline IOP in the present cohort should be interpreted in the context of the study design. Baseline IOP measurements were obtained under ongoing topical glaucoma therapy, and no medication washout was performed. Therefore, baseline values reflected treated preoperative IOP in patients with medically managed mild-to-moderate open-angle glaucoma undergoing combined cataract and MIGS, rather than untreated IOP. This explains the limited overall IOP reduction observed at 60 months. However, the additional exploratory post hoc analysis showed that eyes with higher treated baseline IOP demonstrated greater numerical IOP reduction, whereas eyes with lower treated baseline IOP mainly maintained similar IOP with a reduced medication burden. The medication-sparing effect observed in the Hydrus group is consistent with the long-term results of the HORIZON trial, in which Hydrus implantation combined with cataract surgery provided durable benefit compared with cataract surgery alone [6]. At 5 years, the HORIZON study reported a higher proportion of medication-free eyes and a lower rate of secondary incisional glaucoma surgery in the Hydrus group [6]. Although the present study did not include a phacoemulsification-only control group, our findings similarly support the durability of medication reduction after Hydrus implantation combined with cataract surgery.

Our results are also broadly aligned with the COMPARE study, a prospective randomized trial comparing Hydrus with two iStent devices as standalone MIGS procedures [7]. In COMPARE, Hydrus was associated with higher complete surgical success, greater medication reduction, and a higher proportion of medication-free eyes at 12 months [7]. The design and surgical context of COMPARE differ from the present study, as our cohort underwent combined cataract surgery and MIGS and was followed for 5 years. Nevertheless, both studies support the concept that Hydrus may provide a greater reduction in medication burden than trabecular bypass stenting alone.

Published comparative data are not fully uniform. Holmes et al. reported favorable 24-month outcomes for both Hydrus and iStent inject when combined with cataract surgery, without a significant difference in IOP outcomes between groups [8]. Their real-world registry analysis also suggested differences in medication outcomes that were not identical to those observed in our cohort [8]. Such discrepancies are expected across MIGS studies and may reflect differences in study design, baseline IOP, glaucoma severity, device generation, medication protocols, surgeon selection, and the degree of treatment selection bias. In particular, registry-based studies may be influenced by individualized device choice according to anatomical features, target IOP, perceived need for medication reduction, or surgeon preference.

Other available evidence also supports the role of combined phacoemulsification and MIGS in reducing treatment burden in open-angle glaucoma. Lee et al. showed that combined phacoemulsification and MIGS may provide greater reductions in IOP and glaucoma medications than phacoemulsification alone [9]. In addition, the network meta-analysis by Hu et al. suggested that Hydrus and two-iStent implantation may have a higher probability of achieving medication-free status than single iStent implantation when combined with phacoemulsification [10]. This is relevant to the present study because the comparison involved Hydrus Microstent and first-generation single iStent implantation, both performed at the time of cataract surgery.

The anatomical and functional differences between the two implants may partly explain the stronger medication-sparing effect observed after Hydrus implantation. The first-generation iStent creates a focal trabecular bypass, whereas the Hydrus Microstent acts as an intracanalicular scaffold spanning approximately 90 degrees of Schlemm’s canal and may improve access to multiple collector channels [11]. A broader canal-based mechanism could plausibly reduce the need for topical therapy over time, even when final treated IOP is similar. However, the present data should not be interpreted as proof of mechanistic superiority. The study was not randomized, and treatment allocation may have been influenced by clinical or anatomical factors.

Visual field outcomes were additionally assessed in an exploratory manner in the present revision. Baseline and 60-month MD values were compared when reliable visual field data were available, and eyes with MD deterioration of ≥2.5 dB were identified. This analysis was included to provide clinically meaningful information on functional stability over long-term follow-up. However, the visual field findings should be interpreted cautiously because the number of eyes with complete and reliable long-term perimetric data was limited. Therefore, the MD analysis should be considered supportive and exploratory rather than definitive evidence of between-device differences in glaucoma progression.

Follow-up completeness is another important consideration in interpreting the present results. Five-year IOP, medication, and BCVA data were available for 47 of 65 eyes, corresponding to 72.3% of the original cohort. To address the possibility of attrition bias, baseline characteristics of eyes with available 60-month data were compared with those of eyes without 60-month follow-up data and are presented in Supplementary Table S1. Although this analysis increases transparency, it does not eliminate the potential influence of missing long-term data. In particular, if patients with better disease control were more likely to remain in long-term follow-up, medication outcomes and medication-free success rates may have been overestimated. The results should therefore be interpreted as available-case 5-year outcomes rather than as a complete cohort analysis.

Safety remains central when evaluating MIGS, especially in patients with mild-to-moderate glaucoma undergoing cataract surgery. In the present study, both procedures showed a favorable long-term safety profile. No intraoperative complications were recorded, and no eye required additional glaucoma surgery or selective laser trabeculoplasty over 5 years. Some postoperative findings, including anterior chamber blood cells and peripheral anterior synechiae, were more frequent in the Hydrus group, which may be related to the intracanalicular design of the device and the greater extent of Schlemm’s canal instrumentation. In the Hydrus group, PAS were first documented at the 1-month postoperative visit. They were focal and located near the inlet portion of the implant. No case of implant obstruction was observed, and PAS were not associated with the need for additional glaucoma procedures or laser intervention during follow-up. Importantly, these findings were transient or clinically manageable and did not result in additional surgical intervention during follow-up.

The absence of secondary glaucoma surgery in both groups is clinically reassuring, but it should not be overinterpreted. The cohort was relatively small, baseline IOP was modest, and patients had open-angle glaucoma suitable for combined cataract surgery and MIGS. Therefore, these findings should not be extrapolated to eyes with advanced glaucoma, substantially higher baseline IOP, or very low target pressures. In such cases, more intensive surgical approaches may still be required.

This study has several limitations. First, although the cohort was prospectively followed, the comparison was non-randomized, and treatment allocation may have been influenced by surgeon preference, anatomical considerations, or baseline clinical characteristics. Second, the sample size was modest, particularly at the 5-year visit. Third, analyses were based on available cases rather than imputation of missing data, which may affect the robustness of long-term estimates. Fourth, no medication washout was performed; therefore, the study reflects treated IOP under routine clinical care rather than unmedicated device-specific IOP reduction. In addition, no formal correction for multiple testing was applied. Because several outcomes and follow-up time points were analyzed, the possibility of Type I error should be considered when interpreting secondary and exploratory findings.

Fifth, baseline IOP was relatively low because measurements were obtained under ongoing topical therapy, which limited the potential magnitude of further pressure reduction and made medication burden a particularly relevant endpoint.

Sixth, the availability of long-term visual field data was limited. Finally, the iStent group received the first-generation iStent, and the results should not be directly extrapolated to newer-generation iStent inject or iStent inject W devices.

Despite these limitations, the study adds clinically relevant long-term comparative evidence on canal-based MIGS combined with phacoemulsification. Both first-generation iStent and Hydrus Microstent implantation maintained comparable treated IOP over 5 years. However, Hydrus was associated with a greater reduction in medication burden and a higher rate of medication-free complete surgical success. In clinical practice, this difference may be important when the main therapeutic goal is to reduce dependence on topical glaucoma treatment while maintaining stable IOP control.

## 5. Conclusions

In this 5-year prospective non-randomized comparative follow-up study, first-generation iStent Trabecular Micro-Bypass and Hydrus Microstent implantation combined with phacoemulsification provided comparable long-term treated IOP control in eyes with open-angle glaucoma. Hydrus implantation was associated with a significantly lower medication burden and a higher rate of medication-free complete surgical success using the IOP ≤ 18 mmHg criterion. No eye in either group required additional glaucoma surgery or selective laser trabeculoplasty during follow-up. These findings suggest that, in appropriately selected patients undergoing cataract surgery, Hydrus may offer a greater long-term medication-sparing benefit while maintaining comparable treated IOP; however, this observation should be interpreted cautiously given the non-randomized design and available-case follow-up. Further randomized studies with larger cohorts and complete long-term functional follow-up are warranted.

## Figures and Tables

**Table 1:** Baseline demographic and clinical characteristics of the study groups.

Parameter	iStent N = 35	Hydrus N = 30	*p* Value
Eyes, % right	16 (45.7)	16 (53.3)	0.622 ^a^
Gender, % female	25 (71.4)	24 (80.0)	0.566 ^a^
Age, mean (SD)	72.5 (7.6)	73.4 (11.4)	0.319 ^b^
BCVA, mean (SD)	0.48 (0.23)	0.47 (0.16)	0.759 ^b^
IOP, mean (SD)	16.1 (3.2)	16.4 (2.2)	0.541 ^b^
Number of glaucoma medications, mean (SD)	1.86 (0.94)	1.60 (0.72)	0.327 ^b^
CCT, mean (SD)	530.3 (28.7)	539.3 (42.2)	0.449 ^b^
MD, mean (SD)	−5.79 (3.97)	−6.28 (5.48)	0.957 ^b^
PSD, mean (SD)	4.30 (2.57)	4.38 (2.55)	0.930 ^b^

Data are presented as n (%) or mean (SD). ^a^ Fisher’s exact test or chi-square test; ^b^ Mann–Whitney U test. Abbreviations: BCVA—best-corrected visual acuity; CCT—central corneal thickness; IOP—intraocular pressure; MD—mean deviation; PSD—pattern standard deviation; SD—standard deviation.

**Table 2:** Comparison of mean IOP values before surgery vs. values after 12, 24, 36, 48, and 60 months after surgery.

iStent	*n*	Mean	95% CI	*p* Value
D0	35	16.1	14.96–17.16	
M12	29	15.0	13.87–16.06	
D0–M12	29	1.4	0.37–2.39	0.009
M24	29	15.6	14.47–16.70	
D0–M24	29	0.8	−0.29–1.88	0.145
M36	25	16.0	14.88–17.04	
D0–M36	25	0.6	−0.79–1.99	0.382
M48	25	16.6	15.64–17.48	
D0–M48	25	0.0	−1.35–1.35	1.000
M60	25	16.7	15.95–17.41	
D0–M60	25	−0.1	−1.39–1.15	0.848
**Hydrus**	***n***	**mean**	**95% CI**	***p* value**
D0	30	16.4	15.60–17.27	
M12	22	15.6	14.68–16.51	
D0–M12	22	0.9	−0.51–2.24	0.206
M24	21	16.2	14.87–17.51	
D0–M24	21	0.1	−1.34–1.53	0.891
M36	22	16.2	15.00–17.46	
D0–M36	22	0.2	−1.31–1.77	0.762
M48	22	16.6	15.52–17.67	
D0–M48	22	−0.1	−1.59–1.31	0.847
M60	22	16.5	15.63–17.28	
D0–M60	22	0.0	−1.25–1.25	1.000

*p* values refer to within-group comparisons between baseline and each follow-up visit using the paired Student’s *t*-test or Wilcoxon signed-rank test, as appropriate. Positive values for D0–follow-up differences indicate IOP reduction from baseline; negative values indicate IOP increase. Abbreviations: IOP—intraocular pressure; CI—confidence interval.

**Table 3:** Number of glaucoma medications during the 5-year follow-up.

Follow-Up Visit	iStent N	iStent Mean (SD)	Hydrus N	Hydrus Mean (SD)	*p* Value
Baseline	35	1.86 (0.94)	30	1.60 (0.72)	0.327 ^a^
12 months	28	0.61 (0.92)	22	0.05 (0.21)	0.008 ^a^
24 months	26	0.69 (1.01)	21	0.14 (0.36)	0.043 ^a^
36 months	25	0.88 (0.97)	22	0.18 (0.39)	0.004 ^a^
48 months	25	1.16 (1.03)	22	0.23 (0.43)	0.001 ^a^
60 months	25	1.36 (1.08)	22	0.36 (0.49)	0.001 ^a^

Data are presented as mean (SD). ^a^ Mann–Whitney U test. Abbreviations: SD—standard deviation.

**Table 4:** Surgical success and clinical outcomes at 60 months.

Parameter	iStent N = 25	Hydrus N = 22	*p* Value
Complete success, IOP ≤ 18 mmHg	5 (20.0)	14 (63.6)	0.003 ^a^
Qualified success, IOP ≤ 18 mmHg and ≤ 2 medications	17 (68.0)	20 (90.9)	0.079 ^a^
Complete success, IOP ≤ 15 mmHg	1 (4.0)	5 (22.7)	0.085 ^a^
Qualified success, IOP ≤ 15 mmHg and ≤ 2 medications	6 (24.0)	8 (36.4)	0.524 ^a^
Medication-free eyes	6 (24.0)	14 (63.6)	0.009 ^a^
Reduction of ≥1 glaucoma medication	13 (52.0)	18 (81.8)	0.063 ^a^
Reduction of ≥2 glaucoma medications	6 (24.0)	8 (36.4)	0.524 ^a^
IOP ≤ 18 mmHg	20 (80.0)	20 (90.9)	0.423 ^a^
IOP ≤ 15 mmHg	7 (28.0)	8 (36.4)	0.755 ^a^
IOP reduction ≥20% from baseline	2 (8.0)	1 (4.5)	1.000 ^a^
Additional glaucoma surgery or SLT	0 (0.0)	0 (0.0)	—

^a^ Fisher’s exact test. Abbreviations: IOP—intraocular pressure; SLT—selective laser trabeculoplasty.

**Table 5:** Safety outcomes and postoperative complications.

Adverse Event	iStent N = 35	Hydrus N = 30	*p* Value
Blood cells in anterior chamber	0 (0.0)	12 (40.0)	<0.001 ^a^
IOP increase ≥10 mmHg from baseline	4 (11.4)	2 (6.7)	0.678 ^a^
Peripheral anterior synechiae	0 (0.0)	6 (20.0)	0.007 ^a^
Hypotony, IOP < 6 mmHg	0 (0.0)	1 (3.3)	0.462 ^a^
Corneal edema	0 (0.0)	3 (10.0)	0.093 ^a^
Additional glaucoma surgery or SLT	0 (0.0)	0 (0.0)	—

^a^ Fisher’s exact test. Abbreviations: IOP—intraocular pressure; SLT—selective laser trabeculoplasty.

## Data Availability

The data presented in this study are available on request from the corresponding author due to privacy restrictions.

## Supplementary Materials

The following supporting information can be downloaded at: https://www.mdpi.com/article/10.3390/jcm15135076/s1, Figure S1: Baseline and 60-month mean deviation values in eyes with available reliable visual field data; Figure S2: CONSORT-style flow diagram of patient availability during the 5-year follow-up; Table S1: Baseline characteristics of eyes with and without available 60-month follow-up data.

## Abbreviations

The following abbreviations are used in this manuscript:

BCVA	best-corrected visual acuity;
CCT	central corneal thickness;
CI	confidence interval;
IOP	intraocular pressure;
MD	mean deviation;
MIGS	minimally invasive glaucoma surgery;
POAG	primary open-angle glaucoma;
PSD	pattern standard deviation;
SD	standard deviation;
SLT	selective laser trabeculoplasty
