# Supplementary material for: Five-Year Outcomes of First-Generation iStent Versus Hydrus Microstent Implantation Combined with Phacoemulsification in Patients with Open-Angle Glaucoma: A Prospective Non-Randomized Comparative Study
Source: J Clin Med. 2026 Jun 29;15(13):5076. doi: 10.3390/jcm15135076 (PMC13362729; doi:10.3390/jcm15135076)
Supplement: Supplementary file 1 [file jcm-15-05076-s001.zip › jcm-4369069-supplementary.pdf]

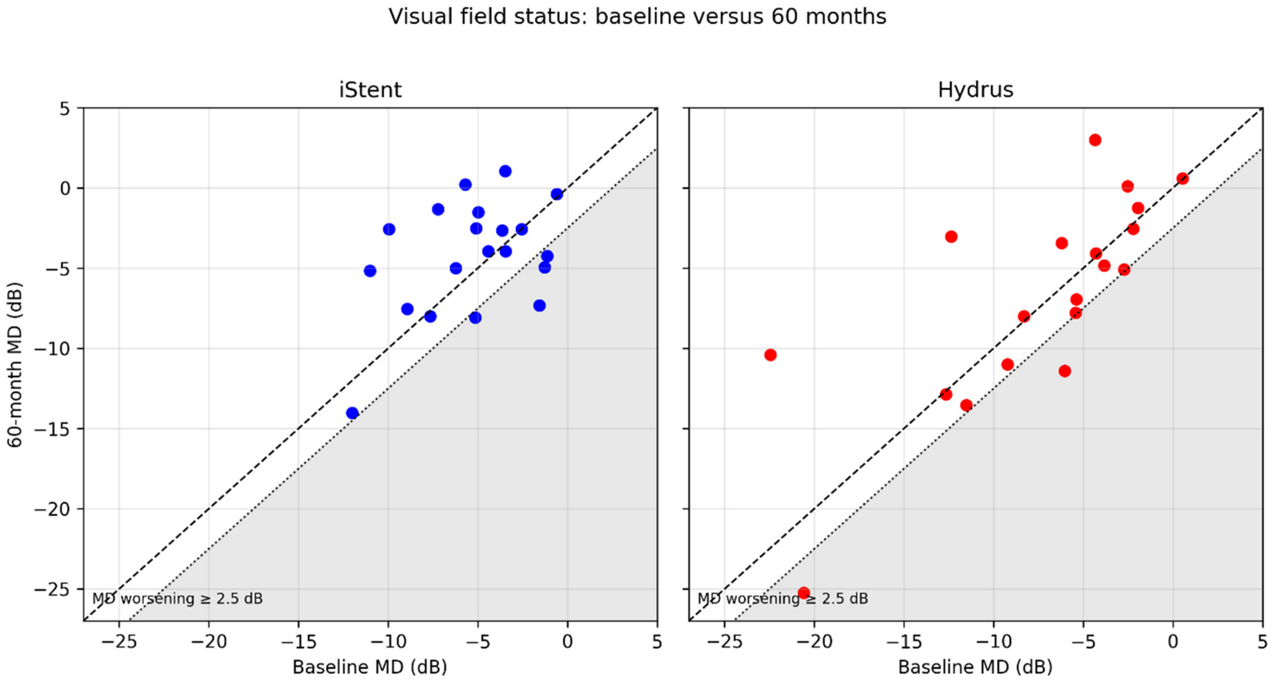

Supplementary Figure S1. Baseline and 60-month mean deviation values in eyes with available reliable visual field data. Eyes with MD deterioration of ≥2.5 dB between baseline and 60 months are indicated.

Scatter plots comparing mean deviation (MD) values at baseline and 60 months in eyes with available visual field data at both time points. The dashed line represents equality between MD values at baseline and 60 months. The dotted line indicates the threshold for MD worsening of ≥2.5 dB relative to baseline. The shaded area represents eyes demonstrating MD worsening of ≥2.5 dB over the 60-month observation period. Among 39 eyes with visual field data available both at baseline and at 60 months, MD worsening ≥2.5 dB was observed in 6 eyes (15.4%), including 4 of 20 eyes (20.0%) in the iStent group and 2 of 19 eyes (10.5%) in the Hydrus group.

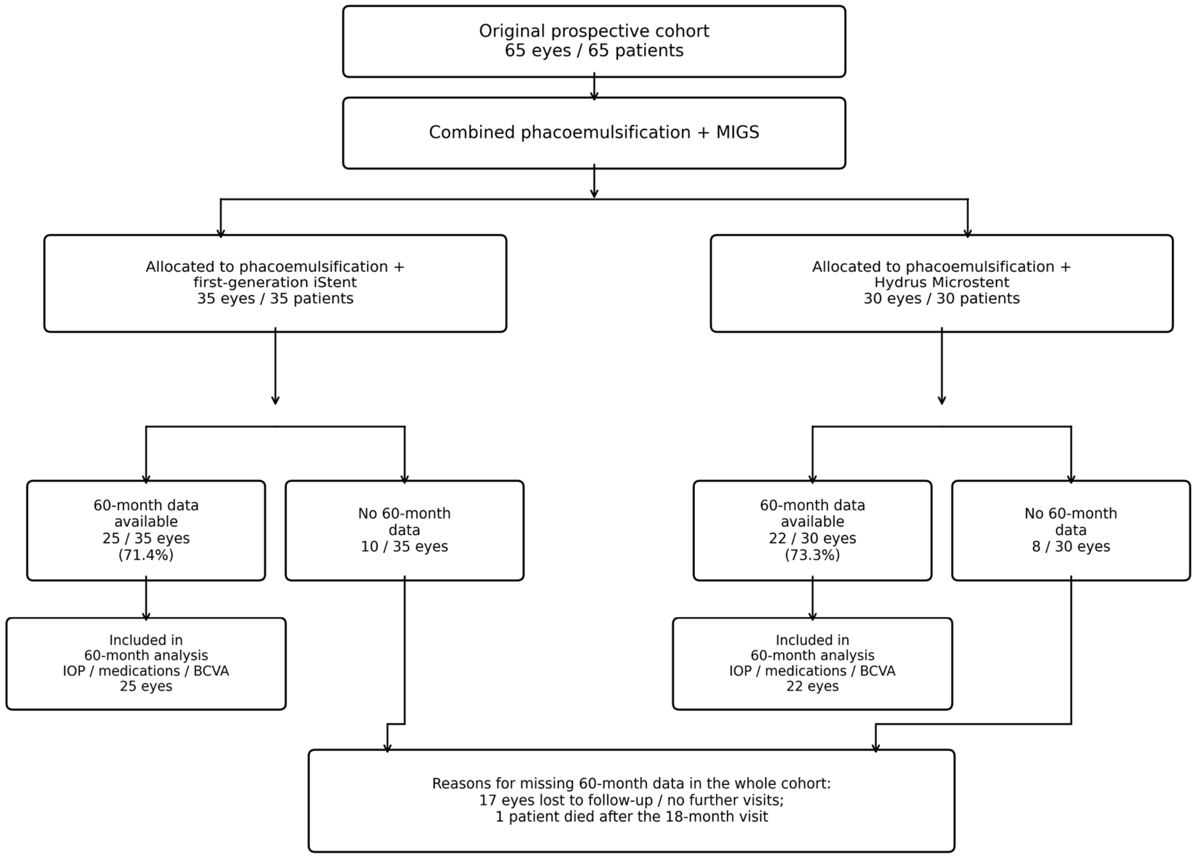

Supplementary Figure S2. CONSORT-style flow diagram of patient availability during the 5-year follow-up.

The diagram shows the number of eyes included in the original cohort, allocated to phacoemulsification combined with first-generation iStent or Hydrus Microstent implantation, and available for the 60-month analysis. Overall, 47 of 65 eyes (72.3%) had available 60-month data, including 25 of 35 eyes in the iStent group and 22 of 30 eyes in the Hydrus group. Seventeen eyes were lost to follow-up because no further visits were recorded, and one patient died after the 18-month visit.

Supplementary Table S1. Baseline characteristics of eyes with and without available 60-month follow-up data.

Parameter	Completers (n = 47)	Non-completers (n = 18)	p-value
Age, years	72.45 ± 10.34	74.24 ± 6.77	0.698
Female sex, n (%)	36 (76.6)	13 (72.2)	0.753
Right eye, n (%)	21 (44.7)	11 (61.1)	0.277
Hydrus implantation, n (%)	22 (46.8)	8 (44.4)	1.000
Baseline IOP, mmHg	16.51 ± 2.80	15.50 ± 2.68	0.249
Baseline glaucoma medications, n	1.81 ± 0.90	1.56 ± 0.70	0.360
Baseline MD, dB	−6.03 ± 4.76	−5.95 ± 4.59	0.861
CCT, µm	534.83 ± 37.29	533.44 ± 31.53	0.860

Data are presented as mean ± SD or n (%).

Abbreviations: CCT—central corneal thickness; IOP—intraocular pressure; MD—mean deviation; SD—standard deviation.
